# Clinical Features and Molecular Characteristics of Methicillin-Susceptible *Staphylococcus aureus* Ocular Infection in Taiwan

**DOI:** 10.3390/antibiotics10121445

**Published:** 2021-11-25

**Authors:** Yueh-Ling Chen, Eugene Yu-Chuan Kang, Lung-Kun Yeh, David H. K. Ma, Hsin-Yuan Tan, Hung-Chi Chen, Kuo-Hsuan Hung, Yhu-Chering Huang, Ching-Hsi Hsiao

**Affiliations:** 1 Department of Ophthalmology, Chang Gung Memorial Hospital, Taoyuan 333, Taiwan; yuehling20@gmail.com (Y.-L.C.); yckang0321@gmail.com (E.Y.-C.K.); lkyeh@ms9.hinet.net (L.-K.Y.); davidhkma@yahoo.com (D.H.K.M.); tanhsin@gmail.com (H.-Y.T.); mr3756@cgmh.org.tw (H.-C.C.); agarlic2000@gmail.com (K.-H.H.); 2 College of Medicine, Chang Gung University, Taoyuan 333, Taiwan; ychuang@cgmh.org.tw; 3 Division of Pediatric Infectious Diseases, Department of Pediatrics, Chang Gung Memorial Hospital, Taoyuan 333, Taiwan

**Keywords:** *Staphyloccus aureus*, MSSA, ocular infection, pulsed-field gel electrophoresis, multilocus sequence typing, Panton-Valentine leukocidin, antibiotic resistance

## Abstract

This study analyzed the clinical features and molecular characteristics of methicillin-susceptible *Staphylococcus aureus* (MSSA) ocular infections in Taiwan and compared them between community-associated (CA) and health-care-associated (HA) infections. We collected *S. aureus* ocular isolates from patients at Chang Gung Memorial Hospital between 2010 and 2017. The infections were classified as CA or HA using epidemiological criteria, and the isolates were molecularly characterized using pulsed-field gel electrophoresis, multilocus sequence typing, and Panton-Valentine leukocidin (PVL) gene detection. Antibiotic susceptibility was evaluated using disk diffusion and an E test. A total of 104 MSSA ocular isolates were identified; 46 (44.2%) were CA-MSSA and 58 (55.8%) were HA-MSSA. Compared with HA-MSSA strains, CA-MSSA strains caused a significantly higher rate of keratitis, but a lower rate of conjunctivitis. We identified 14 pulsotypes. ST 7/pulsotype BA was frequently identified in both CA-MSSA (28.3%) and HA-MSSA (37.9%) cases. PVL genes were identified in seven isolates (6.7%). Both CA-MSSA and HA-MSSA isolates were highly susceptible to vancomycin, teicoplanin, tigecycline, sulfamethoxazole–trimethoprim, and fluoroquinolones. The most common ocular manifestations were keratitis and conjunctivitis for CA-MSSA and HA-MSSA, respectively. The MSSA ocular isolates had diverse molecular characteristics; no specific genotype differentiated CA-MSSA from HA-MSSA. Both strains exhibited similar antibiotic susceptibility.

## 1. Introduction

*Staphylococcus aureus* is a major isolated bacterial pathogen that causes various infections in humans [1]. *S. aureus* is typically categorized as methicillin resistant or methicillin susceptible based on its susceptibility to methicillin. Methicillin-resistant *S. aureus* (MRSA), a strain resistant to all β-lactam antibiotics, warrants particular attention because of its potentially limited treatment options and its increasing prevalence [2]. MRSA has conventionally been considered a health-care-associated (HA) pathogen but has been increasingly reported in community-associated (CA) infections, which is a global health concern [3]. HA- and CA-MRSA strains exhibit distinct clinical presentations, genotypes, and phenotypes [2,4].

Although MRSA has received considerable research attention, methicillin-susceptible *S. aureus* (MSSA) infections are more prevalent than MRSA infections [5]. According to surveillance in eight United States (US) counties in 2016, the incidence of invasive MSSA was 1.8 times higher than that of MRSA; MSSA accounted for 59.7% of HA cases and 60.1% of deaths [5]. In addition, a report published by the Centers for Disease Control and Prevention (CDC) revealed that the incidence of MSSA bacteremia increased by 3.9% annually from 2012 to 2017 in the community in the US [6]. However, few studies have explored the effect of healthcare exposure on the clinical features and molecular typing of MSSA infections [7,8,9,10], potentially due to the clonal diversity of MSSA infections. Most MRSA clones are considered to have evolved from epidemic MSSA clones, leading to the incidence of CA-MRSA [11]. Thus, determination of the genetic characteristics of MSSA strains is crucial for further understanding of the epidemiology of MSSA and even of CA-MRSA.

*S. aureus* is the most common cause of bacterial keratitis (corneal infection) and conjunctivitis (conjunctival infection) [12,13]. Although the Antibiotic Resistance Monitoring in Ocular Microorganisms (ARMOR) study reported that 65.1% of *S. aureus* isolates were MSSA [14], studies on MSSA ocular infections have generally been limited to small case series. Previously, we conducted a 10-year retrospective study on *S. aureus* ocular infections [15] and observed that the percentages of ocular infections caused by MSSA and by MRSA were approximately equal; both strains caused a similar spectrum of diseases, although MRSA was more frequently associated with eyelid infections (16.7% vs. 24.5%, *p* = 0.040). In our subsequent research, we focused solely on MRSA ocular infections [16] and performed molecular typing on MRSA isolates [17]. Because the importance of MSSA should not be overlooked, in this study, we investigated the clinical features, molecular characteristics, and antibiograms of MSSA ocular infections and compared them between CA-MSSA and HA-MSSA isolates.

## 2. Results

### 2.1. Clinical Characteristics of MSSA Ocular Infections

During this 8-year study period, MSSA strains were isolated from 104 patients. Of the 104 patients with MSSA, 46 (44.2%) were classified as having CA-MSSA, and 58 (55.8%) were classified as having HA-MSSA. Table 1 presents a summary of the demographic characteristics and clinical features of the patients with MSSA ocular infections. The patients with HA-MSSA were significantly more likely to have underlying conditions, such as malignancy or current infection, compared with those with CA-MSSA (*p* = 0.006 and <0.001, respectively). Regarding local risk factors, a significantly higher proportion of the patients with HA-MSSA had a history of ocular surgery, and a significantly higher proportion of the patients with CA-MSSA used contact lenses. Keratitis was the most common ocular disease caused by CA-MSSA, followed by conjunctivitis; however, this order was reversed in the HA-MSSA group (Table 2). The rates of keratitis and conjunctivitis caused by the CA-MSSA and HA-MSSA strains were significantly different (keratitis: 63.0% vs. 29.3%, *p* < 0.001; conjunctivitis: 10.9% vs. 39.7%, *p* = 0.001). Most of the patients with CA-MSSA received outpatient or emergency department treatment (69.6%), whereas most of the patients with HA-MSSA infection received inpatient treatment (56.9%).

### 2.2. Molecular Typing

**Figure 1:**
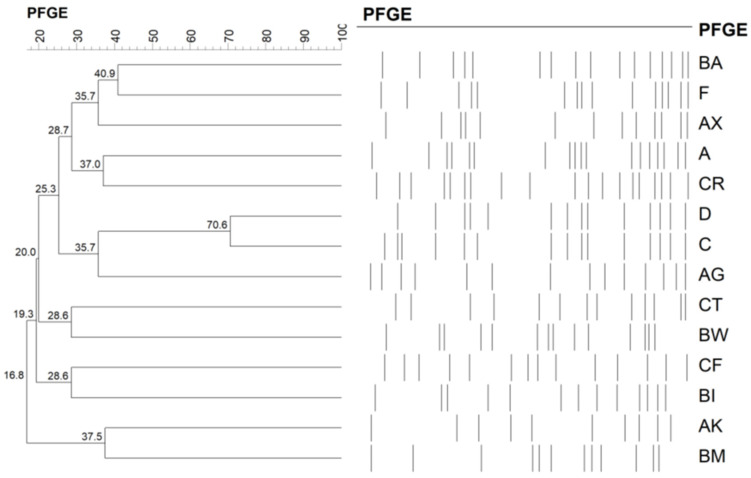
Dendrogram of pulsed-field gel electrophoresis cluster analysis of 104 methicillin-susceptible *Staphylococcus aureus* ocular isolates, classified into 14 pulsotypes.

**Figure 2:**
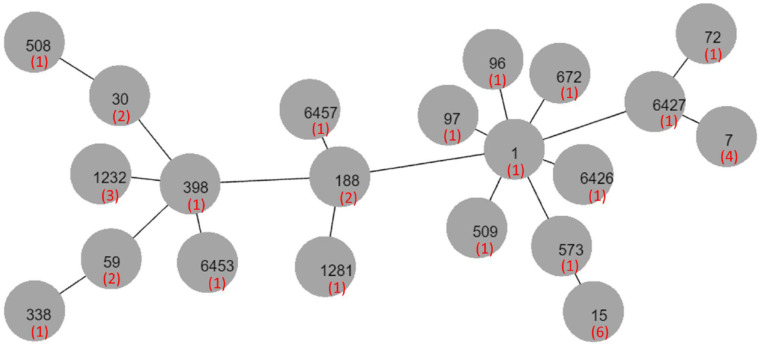
Phylogenic tree of multilocus sequence types of methicillin-susceptible *Staphylococcus aureus* ocular isolates. Bracketed numbers represent the number of isolates.

Table 3 presents the molecular typing of the MSSA isolates. We identified a total of 14 pulsotypes (Figure 1), of which BA was the most common (33.7%), followed by F (19.2%). No specific pulsotype distinguished HA isolates from CA isolates. Multilocus sequence typing (MLST) was performed on 39 isolates selected from each pulsed-field gel electrophoresis (PFGE) type and from four untypeable samples. We identified 21 sequence types (STs); a phylogenic tree of these types is presented in Figure 2. For pulsotype F, all six selected isolates belonged to ST15. For pulsotype BA, cluster complex (CC) 7, including ST7 and ST6427, was the dominant MLST type (5/7). Seven isolates (6.7%), comprising five CA and two HA isolates, contained Panton-Valentine leukocidin (PVL) genes. The PVL-positive isolates contained ST1232 (*n* = 2), ST59, ST338, ST6426, and ST672, as well as one untypeable isolate (Table 3). 

### 2.3. Drug Susceptibility Test

**Figure 3:**
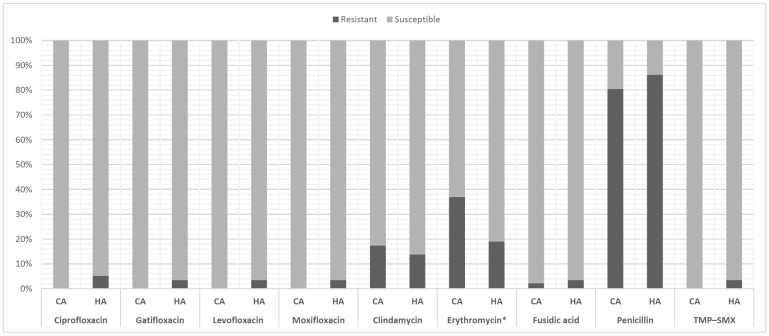
Antibiotic susceptibility of community-associated and health-care-associated methicillin-susceptible *Staphylococcus aureus* ocular isolates. CA = community-associated; HA = health-care-associated; TMP–SMX = sulfamethoxazole–trimethoprim; * *p* = 0.047.

All the CA-MSSA and HA-MSSA isolates were susceptible to linezolid, tigecycline, vancomycin, and teicoplanin. In addition, all the CA-MSSA isolates and more than 95% of the HA-MSSA isolates were susceptible to sulfamethoxazole–trimethoprim (TMP–SMX) and fluoroquinolones. The CA-MSSA and HA-MSSA strains exhibited similar antibiotic susceptibility levels, except for susceptibility to erythromycin (63% vs. 81%, *p* = 0.047) (Figure 3).

## 3. Discussion

Few studies have explored the clinical and molecular characteristics of MSSA infections, particularly ocular infections. In this study, we investigated the clinical and microbiological characteristics of MSSA ocular infections in Taiwan and compared them between CA-MSSA and HA-MSSA ocular isolates, which have rarely been compared in previous studies. We observed that over half of MSSA ocular infections were HA. Keratitis and conjunctivitis accounted for most of the clinical manifestations of the MSSA ocular infections; significantly more patients with CA infections presented with keratitis, whereas those with HA infections more often presented with conjunctivitis. The molecular characteristics of the MSSA isolates were relatively diverse, and no specific genotype differentiated CA-MSSA from HA-MSSA. The antibiograms of the CA-MSSA and HA-MSSA isolates were similar.

In this study, a higher rate of HA-MSSA than of CA-MSSA was observed in the MSSA ocular isolates (55.8%), which is consistent with the findings of our previous 10-year study on *S. aureus* ocular infections [15] but contradicts the findings of a study on pediatric MSSA infections in our hospital in 2015, in which the rate of CA-MSSA was reported to be 71.8% [7]. As expected, patients with HA-MSSA ocular infections exhibited a higher percentage of comorbidities such as malignancy and recent non-ophthalmic infections. Regarding local factors, we observed that a significantly higher proportion of the patients with HA-MSSA infections had undergone ocular surgery; however, a significantly higher proportion of the patients with CA-MSSA infections had a history of contact lens use.

*S. aureus* is the leading cause of bacterial keratitis and is the most common pathogen isolated from patients with conjunctivitis [12,13]. Marangon and Miller studied 1230 MSSA isolates from corneal and conjunctival infections between 1990 and 2001 and observed that the rate of keratitis was 62–65% [18]. Our previous 10-year study [15] and the current study have also confirmed that keratitis and conjunctivitis are common in MSSA ocular infections. In the present study, the patients with CA-MSSA infections exhibited a significantly higher rate of keratitis than did those with HA-MSSA infections, but HA-MSSA isolates caused higher rates of conjunctivitis (followed by keratitis). However, these results are different from those of our previous 10-year report on MRSA ocular infections, in which HA-MRSA isolates caused more keratitis but less conjunctivitis than did CA-MRSA isolates [16].

Regarding molecular characteristics, the MSSA isolates in this study were polyclonal, and none of the clones exhibited significant differences between the CA and HA isolates. Our study revealed that ST7/pulsotype BA, ST15/pulsotype F, and ST188/pulsotype AX accounted for half of the isolates, which is consistent with the findings of previous studies on MSSA nonocular infections in Taiwan [7,10,19]. However, among the samples employed in the previous studies, ST188 was the most common sequence type, whereas in the present study, ST7/pulsotype BA was the most frequent sequence type, which is consistent with the findings of a similar study from China [20]. Although Chen et al. [7] reported that ST15/pulsotype F isolates were more frequently observed in CA-MSSA than in HA-MSSA (*p* = 0.064) among pediatric patients, other research on adult patients [10] did not reveal differences between the genotypes of CA-MSSA and HA-MSSA isolates in Taiwan. Hesje et al. [21] and Peterson et al. [22] have investigated the molecular characteristics of *S. aureus* ocular isolates in the US, and both studies have demonstrated the polyclonality of MSSA ocular isolates. However, Peterson et al. reported that two major USA clonal complexes, CC5 and CC8, were the most frequently detected clones among MSSA and MRSA keratitis isolates. This discrepancy in the molecular characteristics of MSSA ocular isolates indicates geographic variation.

PVL, a bicomponent pore-forming toxin targeting phagocytic leukocytes, is regarded as a marker of CA-MRSA. In this study, we identified PVL genes in seven MSSA isolates (6.7%). The low prevalence of PVL-positive MSSA strains is comparable to that observed in a global clinical trial [8], which reported a rate of 8.8% (118/1334) in MSSA isolates from 2004 to 2005, and to that in a study conducted in central Taiwan, which reported a rate of 5.4% [19]. The endemic CA-MRSA clone might have originated from MSSA. The previous study conducted in Taiwan demonstrated that PVL-positive ST59 MSSA shared a similar genetic profile with PVL-positive ST59 MRSA [23], the endemic CA-MRSA clone. Chen et al. [7] also reported that among pediatric patients at our hospital, 9 of 11 PVL-positive MSSA isolates belonged to ST59. However, the STs of PVL-positive MSSA isolates were heterogenous in this study, with only two belonging to CC59 (ST59 and ST338). Further research is warranted to determine whether the PVL-positive MSSA isolates derived from ocular and nonocular infections are different.

In the present study, the HA-MSSA and CA-MSSA isolates both exhibited high susceptibility to most of the tested antibiotics except for clindamycin and erythromycin. The antibiograms of the MSSA ocular isolates were determined to parallel those of nonocular MSSA isolates in Taiwan. We tested the isolates’ antibiotic susceptibilities to fluoroquinolones, the first-line treatment of ocular infections, and observed that the susceptibility rates to fluoroquinolones all exceeded 90%. This rate is higher than that (approximately 80%) reported by Asbell et al. in their study on MSSA infection in the ocular Tracking Resistance in U.S. Today (ocular TRUST) program [24]. Taiwan’s National Health Insurance Administration strictly regulates the use of ophthalmic fluoroquinolones solutions. Such solutions are reserved for the treatment of severe bacterial infections such as corneal ulcers but are not permitted for prophylactic purposes or the treatment of mild infections, which might have contributed to the relatively high susceptibility of the isolates to fluoroquinolones in our isolates. Although the increasing resistance of MSSA ocular isolates to fluoroquinolones reported by Marangon et al. [18] is a concern, fluoroquinolones might remain the treatment of choice for MSSA ocular infections, at least in Taiwan.

The emergence of drug resistance necessitates innovative therapeutic approaches. Recently, new ophthalmic solutions have been developed. Povidone–iodine 0.6% was determined to exhibit higher bactericidal activity than does povidone–iodine 5% [25]; a commercial solution containing povidone–iodine 0.6% (IODIM, Medivis, Catania, Italy) exhibited high antimicrobial activity against *S. aureus*, *S. epidermidis*, and *Pseudomonas aeruginosa* in vitro [26]. Another solution containing hexamidine diisethionate 0.05% (Keratosept, Bruschettini, Genoa, Italy) also exhibited rapid antibacterial activity against multiresistant *S. aureus*, *S. epidermidis*, and *Candida* species [27]. In addition to bactericidal agents, antibiotic combinations represent an alternative method for treating multiresistant pathogens. Nasir et al. [28] reported that levofloxacin combined with ceftazidime was successful against MRSA isolates. In future research, these treatments should be studied in vivo to determine if they are as effective as they are in vitro.

In contrast to the differentiation of MRSA isolates, differentiating between CA-MSSA and HA-MSSA ocular isolates is difficult. In our previous study on MRSA ocular infections, we found that patients with CA-MRSA ocular infections were, on average, younger and more frequently diagnosed with eyelid disorders than those with HA-MRSA infections [16]. The dominant clones for CA-MRSA and HA-MRSA isolates were ST59 and ST239, respectively [17]; both CA-MRSA and HA-MRSA were multiresistant, but TMP–SMX exhibited high activity against CA-MRSA [16,17]. These results are consistent with those of studies on MRSA nonocular infections in Taiwan. The epidemiological characteristics of each isolate could distinguish CA-MRSA from HA-MRSA isolates and be used to evaluate each isolate’s clinical or pathogenic implications. However, the present study demonstrated that the patients with CA-MSSA and HA-MSSA ocular infections exhibited similar demographic characteristics, microbiological characteristics, and clinical features, except for the spectrum of diseases and admission rates. This implies that the distinction between CA-MSSA and HA-MSSA is blurred, partially due to the transmission of CA strains into HA facilities. Chen et al. [7] investigated the clinical features and molecular characteristics of pediatric MSSA infections in our hospital and determined that more patients with CA-MSSA presented with skin and soft tissue infections than did those with HA-MSSA infections, a clinical feature that is like those of pediatric MRSA infections. The molecular characteristics of the MSSA isolates derived from the pediatric patients were diverse, but one clone (ST15/pulsotype F) exhibited a borderline significant difference between the CA-MSSA and HA-MSSA isolates. Furthermore, the CA-MSSA isolates exhibited a significantly higher susceptibility rate to TMP–SMX (100%) than did the HA-MSSA isolates (95%). Further research involving larger sample sizes is necessary to determine whether the discrepancy between the results of the present study and those of the pediatric MSSA study [7] conducted in the same hospital is due to tissue tropism or other causes.

Our study has several limitations. First, although the isolates were prospectively collected, the clinical data were retrospectively reviewed. Therefore, the patients might have been misclassified due to incomplete evaluation of risk factors. Nevertheless, we were still able to observe some significant differences between the patients infected with the CA and HA-MSSA isolates. Second, the sample size might not have been sufficiently large, but the correlation between pulsotypes and STs is similar to that recorded in the study on pediatric MSSA infection in Taiwan [7]. Third, determining whether the isolate was a contaminant or pathogen was difficult because *S. aureus* is a common colonizer of the ocular surface. However, all the isolates included in this study were clinical samples collected from patients with active ocular infections. Finally, this study was conducted in a single tertiary-care hospital; therefore, the results might not be generalizable to other populations. However, because our hospital is the largest referral hospital in Taiwan, the results of this study may still generally reflect the epidemiology of MSSA infection. Additional prospective studies involving patients from more hospitals are warranted.

## 4. Materials and Methods

### 4.1. Ethics

The study adhered to the tenets of the Declaration of Helsinki and was approved by the Institutional Review Board (IRB) of Chang Gung Memorial Hospital (CGMH), a tertiary medical center in Taoyuan, Taiwan (IRB 107-2346C). The requirement for written informed consent was waived due to the anonymous analysis of the data.

### 4.2. Study Population and Data Collection

From 1 January 2010 to 31 December 2017, clinical *S. aureus* isolates were prospectively collected from patients with ocular infections and stored in the microbiology laboratory of CGMH. The medical records of the patients were retrospectively reviewed for demographic and clinical information. If more than one isolate was collected from a single patient, only the first was included for analysis. The MSSA infections were classified as HA or CA based on the definitions employed by the CDC since 2000 [29]. The clinical criteria for HA-MSSA consist of an infection identified 48 h after hospitalization; a history of healthcare exposure within 1 year prior to the presentation including admission, surgery, dialysis, or residency in a long-term care facility; and the use of permanent indwelling catheters or percutaneous devices. CA-MSSA infections are defined as MSSA infections identified within 48 hours of hospitalization in patients without a history of healthcare exposure within 1 year prior to the presentation.

The patients’ underlying conditions and ocular histories were recorded. The underlying conditions that we screened for were diabetes mellitus, hypertension, pulmonary disease, renal disease, liver disease, malignancy, immunodeficiency, current infection, recent antibiotic use, and alcoholism; we also screened for recent antibiotic use. The ocular histories included contact lens use, ocular surface disease, surgery, and trauma.

Based on the ocular structures involved, the manifestations were categorized into seven diagnoses: lid disorder, conjunctivitis, keratitis, endophthalmitis, wound infection, lacrimal system disorder, and others (including blebitis, buckle or implant infection, and scleral ulcer). If a patient was diagnosed with more than one ocular infection, the primary pathology or the most severe diagnosis was chosen.

### 4.3. Molecular Characteristics

Molecular analysis methods employed in this study included PFGE by SmaI digestion, PVL gene detection, and MLST, with approaches consistent with those described in detail by Lina et al. [30] and Enright et al. [31]. All the MSSA isolates were molecularly characterized on the basis of PFGE and PVL genes. MLST was performed on selected isolates with representative PFGE patterns. The ST was determined according to each isolate’s allelic profile.

### 4.4. Drug Susceptibility Test

We evaluated the antimicrobial susceptibility of the MSSA isolates to antibiotics (namely penicillin, oxacillin, TMP–SMX, clindamycin, erythromycin, fusidic acid, teicoplanin, vancomycin, tigecycline, and linezolid) through the disk diffusion method according to the Clinical and Laboratory Standards Institute’s standards for antimicrobial susceptibility testing [32]. An E test (bioMerieux, Marcy-I’Etoile, France) was also used to determine the isolates’ susceptibility to fluoroquinolones, namely ciprofloxacin, levofloxacin, gatifloxacin, and moxifloxacin. Oxacillin was used instead of methicillin to test for β-lactam resistance.

### 4.5. Statistical Analysis

All statistical analyses were performed using SPSS (Version 19.0; IBM, Armonk, NY, USA). The nominal variables were analyzed through a chi-square test, and the continuous variables were analyzed using Student’s t test. The variables are presented either as a mean ± standard deviation or as a percentage. The correlation between clinical presentations and the classification of MSSA infections as HA or CA was measured using Fisher’s exact test. A two-tailed *p* value of less than 0.05 was considered statistically significant.

## 5. Conclusions

More than half of the MSSA ocular infections included in this study were classified as HA-MSSA. CA-MSSA caused a higher rate of keratitis than did HA-MSSA, whereas HA-MSSA caused a higher rate of conjunctivitis. Although the molecular characteristics of the MSSA isolates indicated that the isolates were genetically diverse, ST7, ST15, and ST188 were frequently observed in the MSSA ocular infections. Both CA- and HA-MSSA strains exhibited high susceptibility to fluoroquinolones in Taiwan. Physicians should be familiar with the epidemiology, spectrum of diseases, and antibiotic susceptibility patterns of MSSA ocular infections in their local areas. Although we could not clearly differentiate HA-MSSA from CA-MSSA in this study, the results provide information that may be used to enhance local public health policy as well as knowledge on epidemic MSSA clones worldwide. Further research should include larger sample sizes and involve more hospitals to deepen the understanding of the molecular characteristics and clinical features of MSSA ocular infections.

## Figures and Tables

**Table 1:** Comparison of demographics and systemic and local factors between patients infected with community-associated and health-care-associated methicillin-susceptible *Staphylococcus aureus* ocular isolates.

	CA (*n* = 46) No. (%)	HA (*n* = 58) No. (%)	*p* Value
**Demographics**			
Age in years, mean ± SD (range)	44.6 ± 24.0 (0.1–84)	52.6 ± 25.0 (0.1–95)	0.102
Sex (male/female)	22/24 (47.8/52.2)	25/33 (43.1/56.9)	0.694
**Underlying Condition**			
Diabetes mellitus	9 (19.6)	15 (25.9)	0.491
Hypertension	13 (28.3)	20 (34.5)	0.532
Pulmonary disease	3 (6.5)	6 (10.3)	0.728
Renal disease	4 (8.7)	6 (10.3)	1
Liver disease	2 (4.3)	5 (8.6)	0.46
Malignancy	1 (2.2)	12 (20.7)	**0.006**
Immunodeficiency	1 (2.2)	5 (8.6)	0.224
Current infection ^a^	0 (0)	19 (32.9)	**<0.001**
Recent antibiotic use	1 (2.2)	15 (25.9)	**0.001**
Alcoholic	2 (4.3)	3 (5.2)	1
**Ocular history**			
Contact lens use	8 (17.4)	1 (1.7)	**0.010**
Ocular surface disease	11 (23.9)	23 (39.7)	0.098
Surgery	9 (19.6)	37 (63.8)	**<0.001**
Trauma	8 (17.4)	9 (15.5)	0.797

^a^ Nonocular infection; CA = community-associated; HA = health-care-associated.

**Table 2:** Comparison of diagnoses and treatments between community-associated and health-care-associated methicillin-susceptible *Staphylococcus aureus* ocular isolates.

	CA (*n* = 46) No. (%)	HA (*n* = 58) No. (%)	*p* Value
**Diagnosis**			
Lid disorder	4 (8.7)	4 (6.9)	0.730
Conjunctivitis	5 (10.9)	23 (39.7)	**0.001**
Keratitis	29 (63.0)	17 (29.3)	**<0.001**
Endophthalmitis	3 (6.5)	2 (3.4)	0.653
Wound infection)	1 (2.2)	5 (8.6)	0.224
Lacrimal system disorder	4 (8.7)	4 (6.9)	0.730
Others (%)	0 (0)	3 (5.2)	0.253
**Treatment**			
Surgical intervention	5 (10.9)	11 (19)	0.288
Inpatient	14 (30.4)	33 (56.9)	**0.009**
Outpatient/ED	32 (69.6)	25 (43.1)	**0.010**

CA = community-associated; ED = emergency department; HA = health-care-associated.

**Table 3:** Molecular characteristics of 104 methicillin-susceptible *Staphylococcus aureus* ocular isolates stratified by pulsotype.

Pulsotypes	BA	F	AX	BW	D	Others
No. isolates (*n* = 104)	35 (33.7%)	20 (19.2%)	8 (7.7%)	8 (7.7%)	7 (6.7%)	26 (25%)
CA (*n* = 46)	13 (28.3%)	10 (21.7%)	4 (8.7%)	3 (6.5%)	5 (10.9%)	11 (23.9%)
HA (*n* = 58)	22 (37.9%)	10 (17.2%)	4 (6.9%)	5 (8.6%)	2 (3.4%)	15 (25.9%)
*p*-value	0.404	0.621	0.730	1	0.237	1
PVL-positive (*n* = 7)	0	0	1	1	2	3
Sequence type	1 (1/7), 7 (4/7), 6427 (1/7), 6457 (1/7)	15 (6/6)	188 (2/3), 6426 ^a^ (1/3)	Untypeable ^a^ (4/4)	59 ^a^ (2/4), 97 (1/3), 338 ^a^ (1/4)	1232 ^aa^ (3), 59, 1281, 72, 30 (2), 96, 398, 508, 509, 573, 672 ^a^, 6453, untypeable

^a^: Sequence type of PVL-positive isolates; ^aa^: Two isolates with PVL were ST 1232; CA = community-associated; HA = health-care-associated; PVL = Panton-Valentine leukocidin genes.

## Data Availability

The data presented in this study are available upon request.
